# Effect of sevoflurane pretreatment in relieving liver ischemia/reperfusion-induced pulmonary and hepatic injury^1^

**DOI:** 10.1590/s0102-865020190080000005

**Published:** 2019-10-14

**Authors:** Guiping Xu, Xiaoli Wang, Yuxiang Xiong, Xueping Ma, Li Qu

**Affiliations:** I Professor, Department of Anesthesia, Xinjiang Uygur Municipal People’s Hospital, Urumqi 830001, China. Conception, design, intellectual and scientific content of the study; manuscript writing; critical revision; final approval.; II MD, Department of Anesthesia, Xinjiang Uygur Municipal People’s Hospital, Urumqi 830001, China. Acquisition and analysis of data, manuscript writing.; III MD, Department of Anesthesia, Xinjiang Uygur Municipal People’s Hospital, Urumqi 830001, China. Acquisition and analysis of data.

**Keywords:** Sevoflurane, Ischemia, Reperfusion, Liver, Lung Injury, Rats

## Abstract

**Purpose:**

To investigate the effect of sevoflurane preconditioning on ischemia/reperfusion (I/R)-induced pulmonary/hepatic injury

**Methods:**

Fifty-one Wistar rats were randomly grouped into sham, I/R, and sevoflurane groups. After reperfusion, the structural change of the lung was measured by Smith score, the wet and dry weights (W/D) were determined, malondialdehyde (MDA) myeloperoxidase (MPO) content was determined colorimetrically and by fluorescence, respectively, and matrix metalloprotein-9 (MMP-9) mRNA was quantified by RT-PCR. Biopsy and morphological analyses were performed on liver tissue, activities of aspartate aminotransferase (AST) and alanine aminotransferase (ALT) were determined, and tumor necrosis factor-alpha (TNF-α) level was determined.

**Results:**

The sham group showed no changes in tissue structure. Structural lesions in the sevoflurane and I/R groups were mild and severe, respectively. Smith score, W/D, MDA, MPO, and MMP mRNA showed the same trend, and were increased in the I/R group and recovered in the sevoflurane group, compared with the sham group (both P<0.05). AST and ALT were significantly increased compared to the sham group (AST: 655±52.06 *vs* . 29±9.30 U/L; ALT: 693±75.56 *vs* . 37±6.71 U/L; P<0.05). In the sevoflurane group, AST and ALT levels were significantly decreased (464±47.71 and 516±78.84 U/L; P<0.001). TNF-α presented similar results.

**Conclusion:**

The protection of lung and liver by sevoflurane may be mediated by inhibited leukocyte recruitment and MMP-9 secretion.

## Introduction

Systemic inflammatory responses (SIRS) are one of the main risk factors after liver transplantation^1^ , can be caused by hepatic ischemia/reperfusion (I/R) injury^2^ , and their manifestation includes neutrophil infiltration, edema, pulmonary alveoli, inner hemorrhage, and endothelial cell activationin^3^ .

In hepatic I/R injury^4 , 5^ , matrix metalloproteinases (MMPs) often increase because of ischemia-induced oxidative stress and cytokines^8 , 9^ , which would lead to further liver damage, like hepatic cell variation, and matrix structure remodeling^6^_._ In addition, tumor necrosis factor-alpha (TNF-α) is involved in the destruction of the extracellular matrix, and may increase MMP-9 levels^7^ .

Sevoflurane is a volatile anesthetic with the unique clinical characteristic of rapid recovery time and relatively low risk. It is suitable for both the induction and maintenance of anesthesia^8^ , and sevoflurane preconditioning has a protective effect during liver I/R-induced pulmonary injury^9 - 12^ .

In the present study, we evaluated if there is a direct beneficial relationship between MMP-9/TNF-α and sevofluranein I/Rinjury^13 , 14^ . This study aimed to evaluate the protective effect of sevoflurane preconditioning on liver I/R-induced pulmonary and hepatic injury.

## Methods

This study was carried out in strict accordance with the recommendations in the Guide for the Care and Use of Laboratory Animals of the National Institutes of Health. The animal use protocol was reviewed and approved by the Institutional Animal Care and Use Committee (IACUC) of the Medical University. Fifty one healthy adult male Wistar rats weighing 240 to 330 g were provided by the Experimental Animal Center of the Medical University.

###  Preparation of I/R-induced pulmonary injury model 

As previously described ^13^ , the rats were used to prepare a 70% liver I/R-induced pulmonary injury model. Rats were anesthetized by intraperitoneal injection of 2% sodium pentobarbital at an initial dose of 80 mg/kg and were fixed in the supine position on a thermic operating table. Under direct visualization, the hepatic portal vein and hepatic artery were clamped with a micro-vessel clip for 30 min to induce liver left and middle lobe ischemia within 0.5 min. Compared to the unblocked right lobe, the blocked left lobe of the liver became notably white, indicating that the blocking was successful. After 30 min, the abdomen was re-opened to remove the clip, and then the abdomen was closed again for liver reperfusion for 60 min. During I/R, the rectal temperature of the animals was measured and maintained at 37.0±0.5°C using the thermic operating table. The rats of the sham group (n=17) were subjected to sham operations without IR injury. All rats were mechanically ventilated (21% O_2_+69% N_2_). By connecting the air inlet of the respirator and circuit end of the anesthesia machine containing sevoflurane, the sevoflurane preconditioning group of rats (n=17) was pretreated with the ventilator-assisted inhalation of 2% sevoflurane (Ohmeda 210, Datex-OhmedaInc., Madison, WI, USA) in 21% O_2_) (Chengdu Taimeng Technology Co., Ltd., Chengdu, China) 30 min before induction of IR injury. After reperfusion, blood samples were immediately collected for centrifugation, serum separation, and use for biochemistry analysis. At the end of reperfusion, 4% paraformaldehyde was injected into the trachea to maintain left lung expansion at a sustained 25 cm water column pressure to prepare the lung histological sections, which were then evaluated by determining the Smith score.^15^ Pulmonary edema, infiltration of alveolar and interstitial inflammatory cells, alveolar and interstitial hemorrhage, atelectasis, and formation of hyaline membrane were used to assess the severity of lung injury using the scoring system. In the scoring system, 0 indicated normal pulmonary vessels, alveoli, interstitium, and bronchi; 1 indicated lesion area was <25% of the whole visual field area; 2 indicated lesion area was 25–50% of the whole visual field; 3 indicated lesion area was 50–75% of the whole visual field; and 4 indicated lesion area was >75% of the whole visual field. The total lung injury score was 4 points. The total points were the sum of the above parameters (21% O_2,_ 5 L/min). The right lung was frozen in liquid nitrogen and stored at −80°C for determining malondialdehyde (MDA) and myeloperoxidase (MPO) levels, and for performing RT-PCR.

###  Lung histology 

After tissue preparation, part of the −80°C stored lung tissue was homogenized to prepare a 10% tissue homogenate, which was used to detect MDA by a colorimetric method. The wet/dry (W/D) weight was calculated with dry weight (65°C, 24 hours)/wet weight of the lung. Two evaluators participated in this analysis. Ten fields were analyzed for each sample and the magnification of visual field was ×200. The pathological results of lung tissue were observed by light microscopy and the Smith score was used to semi-quantitatively evaluate pulmonary edema, alveolar and interstitial inflammation, alveolar and interstitial hemorrhage, atelectasis, and hyaline membrane formation. In the scoring system, 1 indicated a lesion range <25%; 2, 25% to 50%; 3, 50% to 75%; and 4: >75%. The final lung injury score was the average.

###  Biochemical analysis 

A model 2700 automatic biochemical analyzer (Olympus, Tokyo, Japan) was used to measure alanine aminotransferase (ALT) and aspartate aminotransferase (AST) to assess liver injury. Serum tumor necrosis factor-alpha (TNF-α) was assessed by double-antibody sandwich enzyme linked immunosorbent assay (E-EL-R0019c; Elabscience, Houston, TX, USA). MPO activity was detected with a fluorescence detection kit (Cayman Chemical, Ann Arbor, MI, USA). Since MPO is a specific enzyme with a stable proportion in neutrophils, the detection of MPO activity could reflect the degree of infiltration and number of central granulocytes in tissues. MPO reacts with hydrogen peroxide, and then reacts with the complex and hydrogen donor to obtain water and MPO, as well as the final product. The colors of the reactants were detected, and the absorbance was detected using a spectrophotometer, so that the content of active MPO and corresponding neutrophils could be obtained. The content of MDA in lung tissue was detected by colorimetry. Before the detection, the standard curve was drawn based on a standard sample diluted to 1, 2, 5, 10, 20, and 50 µL with distilled water. Each sample was centrifuged and the MDA in the recovered supernatant was measured at 532 nm.

###  RT-PCR 

**Figure 1:**
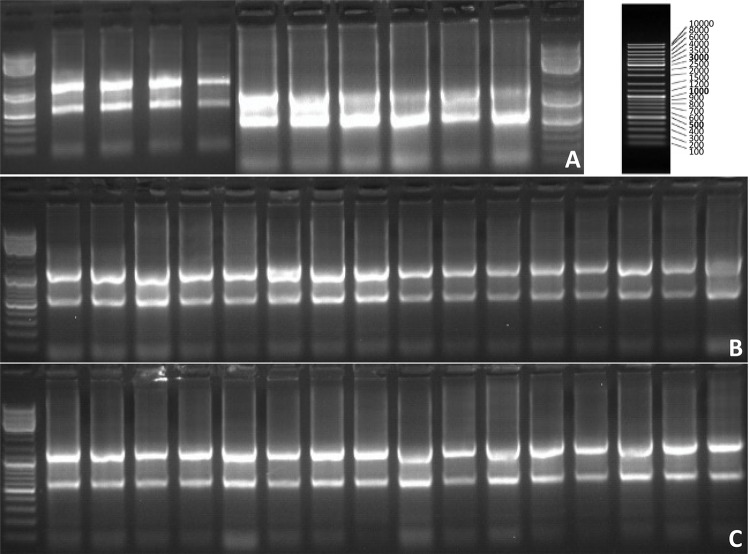
PCR gel electrophoresis patterns in the sham operation group (A), I/R group (B), and sevoflurane group (C).

MMP-9 was detected by reverse transcription-polymerase chain reaction (RT-PCR). Total RNA was extracted from the frozen lung tissue of the 51 samples and reverse-transcribed into cDNA ( Table 1 ). The following primers were used for PCR: MMP-9 forward primer 5′-CCCTGCGTATTTCCATTCATC-3′, reverse primer 5′-AACCATCCGAGCGACCTTT-3′, length 73 base pairs; internal control β-actin forward primer 5′-CGTAAAGACCTCTATGCCAACA-3′, reverse primer 5′-AGCCACCAATCCACACAGAG-3′, length 163 base pairs. Five microliters of the PCR product were added in 2% agarose gel for gel electrophoresis, and the band was measured by optical densitometry. The concentration of MMP-9 was determined using 2^-^( Fig. 1 ).

**Table 1:** The provider of agents during PCR.

Agents	Pharmacy
Trizol	Aidlab
Hiscript Reverse Transcriptase (RNase H)	VAZYME
5xHiScript Buffer	VAZYME
DdH_2_O (DNase/RNase Free)	genecopoeia
Ribonuclease Inhibitor	TRANS
dNTP	TIANGEN
50xROX Reference Dye 2	VAZYME
SYBR Green Master Mix	VAZYME
Taq Plus DNA Polymerase	TIANGEN
DNA Marker	TIANGEN
Randam primer	AIDLAB
Primer	Tinayi Huiyuan

###  Statistical analysis 

Data were analyzed using SPSS 16.0 software (SPSS, Inc., Chicago, IL, USA) and expressed as mean±standard deviation (mean±SD). Differences between and within groups were compared by analysis of variance with least significant difference as post-hoc analysis. A *P-* value < 0.05 was considered significant.

## Results

###  Lung histology 

**Figure 2:**
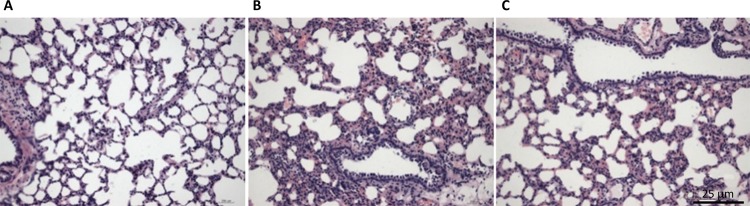
The pulmonary injury was severe in the I/R group (B) and moderate in the sevoflurane group (C) compared to the sham group (A).

In the comparison of the pulmonary injury scores for the sham operation, I/R, and sevoflurane groups, the semi-quantitative evaluation of pulmonary injury revealed a significant difference in the degree of pulmonary injury. The severity of lung injury in animals receiving I/R was reduced in the sevoflurane group ( Table 2 , Fig. 2 ).

**Table 2:** Smith score of different groups.

Sham (n=17)	I/R (n=17)	Sev (n=17)
1.12±0.04	2.15±0.36	1.90±0.34

###  Liver I/R injury 

Compared with the sham group, serum AST and ALT levels were significantly increased in the I/R group, whereas AST and ALT, and TNF-α levels in the sevoflurane group were lower than those in the I/R group ( Table 3 ).

**Table 3:** Serum AST/ALT/TNF-α level in different groups.

	Sham (n=17)	I/R (n=17)	Sev (n=17)
AST (U/L)	29±9.30	655±52.06*	464±47.71*^#^
ALT(U/L)	37±6.71	693±75.56*	516±78.84*^#^
TNF-a (ng/ml)	4.07±0.13	5.36±0.28*	4.45±0.25*^#^

Serum AST and ALT level in performing reperfusion rats: Compared with sham group: ^*^ *P* <0.05, compared with I/R group: ^#^ *P* <0.05.

###  Biochemical analysis 

The W/D weight increased in the I/R group. MDA and MPO levels were also increased, but were down-regulated by sevoflurane. Additionally, the MMP9 mRNA level in the I/R group was significantly higher than that in the sham group, which was also reduced by sevoflurane. MMP-9 (Gelatinase B) activity was assessed to evaluate MMP activity in the partial liver and lung following I/R-induced pulmonary injury, which may induce matrix degradation.

MPO activity is indirect evidence of neutrophil infiltration. The mean MPO activity in the I/R group was higher than that in the sham operation group, while in the sevoflurane group, it was lower. Acute liver I/R was associated with MMP-9 activation ( Table 4 ).

**Table 4:** The differences of W/D, MDA, MPO and MMP-9 mRNA of lung tissue in different groups.

	Sham (n=17)	I/R (n=17)	Sev (n=17)
W/D (%)	3.51±0.12	4.70±0.43*	4.43±0.24*^#^
MDA (nmol/mg)	0.53±0.10	0.88±0.18*	0.74±0.12*^#^
MPO (U/g)	0.82±0.06	1.79±0.24*	1.49±0.13*^#^
MMP-9mRNA	0.84±0.66	3.95±0.13*	3.46±0.15*^#^

Compared with sham operation group: *P<0.05, Compared with I/R group: #P<0.05.

In I/R group, MPO and MMP-9 mRNA were higher when compared with Sham (MPO: 1.79±0.24 U/g vs. 0.82±0.06 U/g; MMP-9 mRNA: 3.95±0.13 *vs* . 0.84±0.66; P<0.05), while in Sev group the level of both MPO and MMP-9 mRNA were lower down (MPO: 1.49±0.13 U/g MMP-9 mRNA: 3.46±0.15).

## Discussion

I/R injury characterized by robust sterile inflammatory responses remains a challenge in diverse clinical situations, such as organ transplantation, thromboembolic events, and cardiac arrest^16^ . Due to dual blood supply systems and continuous physiological demand for oxygen uptake and gas exchange of the lung, the liver and lung appeared particularly vulnerable to I/R injury. In addition, the molecular mechanisms underlying I/R injury of liver and lung are thought to be more complicated than those in other organs^17 , 18^ . Lungs induced response of I/R injury^8 , 19^ , which related to proinflammatory cytokines, oxygen-derived radicals, and activated neutrophils and cause a systemic inflammatory response, which led to other organ injury^17^ . I/R injury represents a potentially maladaptive response of the innate immune system, which features an exacerbated sterile inflammation response triggered by tissue damage^20 , 21^ .

From our study, we’ve found that sevoflurane could relieve liver I/R by inhibiting oxygen-derived radicals^20^ and lipid oxidative reactions^22^ . Although the protective mechanisms of pharmacological conditioning involving sevoflurane have not been fully understood, the endothelial glycocalyx in the liver tissue is preserved against ischemia-reperfusion injury, and appears to involve multiple pathways that may be initiated before ischemia (preconditioning) or during reperfusion (post-conditioning)^23^ . Presently, serum AST and ALT levels were higher in the sevoflurane group than in the sham group, but lower than in the I/R group, indicating that sevoflurane preconditioning relieves liver I/R injury. Also the pulmonary tissue injury scores of sevoflurane group was lower than the I/R group, which were consistent in both liver and lung. In all three groups, pulmonary W/D values were significantly increased because of increased capillary permeability. The content of MPO, a PMN infiltration biomarker, was increased. In acute graft injury and after liver transplantation, sevoflurane has the same effect on I/R injury compared to propofol^24^ and the pretreatment of sevoflurane appears to help protect hepatocytes against I/R-induced necrosis^25^ .

MMP-9 activity was increased in pulmonary tissue with acute liver I/R injury, and this was associated with increased serum TNF-α and injury to the pulmonary tissue. MMP-9 is a member of the MMP family, which can break down the extracellular matrix by inducing the production of proinflammatory cytokines including interleukin-1β, and MMP-9 stored in the tertiary particles of PMNs, which is crucial in acute inflammatory disease^26^ . Although the mechanism remains unclear, the increase in MMP-9 activity may be related to the high level of TNF-α, indicating liver I/R injury and remote lung injury. We observed higher TNF-α level and MMP-9 mRNA relative expression value in the I/R and sevoflurane groups compared to the values in the sham group, indicating that the effects of sevoflurane on pulmonary injury may be related to the reduction of serum TNF-α and down-regulation of MMP-9 mRNA expression. A previous study demonstrated that MMP can regulate cytokines in septicemia-induced pulmonary injury by controlling platelet-secreting CD40Lprotein^27^ . Additionally, MMP-9 can actively promote activation of MPO, which is an index of neutrophil infiltration^28^ . Once inflammation occurs, neutrophils are the first batch of cells recruited to the injury or inflammatory site^29^ . After liver I/R injury, MPO activity increases in ischemic liver tissue. Occasionally, a few neutrophils and/or small granulomas were observed surrounding the necrotic cells in the ischemic liver lobe. Interestingly, a similar MPO level increase was detected in pulmonary tissue, which was associated with the presence of many granulocytes.

The collective findings indicate that anesthesia established by sevoflurane preconditioning lessens pulmonary injury, protects lung tissue, and relieves injury that has occurred through several underlying mechanisms.

There were a few limitations to this study. Due to the species difference between human and rats, there is limited evidence of sevoflurane protection of I/R injury of the lung and liver. The length of the study was insufficient for the development of serious side effects. Additionally, the mean values of the evaluation index were insufficient to evaluate lung and liver function. Finally, the small sample size and strict drug usage prevent the extension of the results to the clinical situation.

In summary, after I/R injury, MMP-9 activity was increased in remote organs, whereas MMP-9 activity was reduced after sevoflurane preconditioning. This may be useful as a target for evaluating the MOF-associated mechanism and inhibiting MPO progression. In the clinic, sevoflurane inhalation may prove to be a choice in liver transplantation to protect both the liver and lungs. However, more random controlled studies are necessary.

## Conclusion

The protection of lung and liver by sevoflurane may be mediated by inhibited leukocyte recruitment and MMP-9 secretion.

## Footnotes

1 Research performed at Department of Anesthesia, Xinjiang Uygur Municipal People’s Hospital, Urumqi 830001, China.
